# Publication audit, Editor’s Triage and Responsibilities of the Editors

**DOI:** 10.12669/pjms.291.3373

**Published:** 2013

**Authors:** Shaukat Ali Jawaid

With authors under pressure to publish, increasing scientific misconduct, authorship issues^1^ publishing and maintaining standard of peer review journal has become a lot more complex. Editors in developing Third World countries having financial and human resource constraints coupled with lack of good quality reviewers are faced with enormous problems, in fact it has become a very stressful and frustrating job. ^2^^.^^3^^,^^4^ Editors are also supposed to do their best and play their role in improving standard and quality of their journals despite all these difficulties and problems, trying to get best out of each and every member of the Editorial Board, which of course is not an easy task. ^5^

 We in Pakistan Journal of Medical Sciences have been trying to increase visibility of the journal to attract more authors and increase in readership from countries in Asia-Pacific Region in particular. We follow an author friendly policy, try to help and guide the authors to improve their manuscripts sometimes re-writing a significant portion of their manuscripts, improving English language and Grammar where needed. However, at times some of the submissions are “dead on arrival” hence such authors cannot be helped. Even then we do try to point out the major shortcomings, deficiencies as we believe the job of an Editor is of a Mentor rather than imposing punishments.

**Table-I:** Manuscript received by Pak J Med Sci (2007 – 2012)

*Country*	*2007*	*2008*	*2009*	*2010*	*2011*	*2012*
Armenia	-	-	-	-	-	01
Australia	-	-	01	01	-	-
Austria	-	-	-	01	-	-
Bangladesh	07	06	07	10	10	06
Bahrain	-	01	-	-	-	01
Brunei	01	-	-	-	-	-
Cameroon	-	-	02	-	-	-
Canada	-	-	-	-	01	-
China	-	01	-	29	64	189
Cyprus	-	-	-	-	-	01
Czechosalvkia	-	-	-	-	-	01
Egypt	-	-	01	02	01	-
France	-	-	-	-	02	-
Germany	-	-	-	01	-	01
India	21	06	10	17	20	16
Ireland	-	02	-	-	-	-
Iran	149	169	170	262	292	250
Iraq	02	04	09	06	05	13
Jordan	10	04	09	04	06	05
Kuwait	02	-	01	-	02	-
Malaysia	01	03	04	09	08	14
Moroccoo	-	-	-	-	01	02
Nepal	-	-	-	02	01	-
New Zealand	-	-	-	-	01	-
Nigeria	32	34	33	31	16	22
Oman	01	-	01	01	01	06
Pakistan	98	123	146	136	205	170
Palestine	05	04	05	03	04	01
Poland	01	01	01	-	-	-
Romania	-	-	01	-	-	-
Saudi Arabia	11	21	20	14	32	38
South Africa	01	03	-	06	-	05
South Korea	-	02	03	02	04	02
Sudan	-	-	02	03	02	03
Syria	-	-	-	01	-	-
Thailand	-	01	01	-	-	-
Taiwan	-	-	-	03	09	02
Tunisia	-	01	03	-	-	-
Turkey	05	34	80	187	235	265
UAE	-	04	03	02	04	02
Uganda	-	-	-	-	-	02
USA	02	-	01	01	01	-
UK	06	05	01	05	04	05
West Indies	-	-	-	01	-	-
Total (44)	354	427	498	740	931	1023

**Table-II:** General statistics for the Year 2012

Total new submissions	1023
Total number of manuscripts rejected	627
Number of manuscripts published	236
Manuscripts published from Pakistan	65
Manuscripts withdrawn by authors	16
Manuscripts rejected due to plagiarism	28

**Table-III:** Manuscripts Published by Pak J Med Sci (2007–2012)

*Country*	*2007*	*2008*	*2009*	*2010*	*2011*	*2012*
Australia	-	-	01	-	-	-
Bangladesh	05	05	04	04	04	04
Bahrain	-	-	01	01	-	-
Brunei	01	-	-	-	-	_
Cameroon	-	-	-	01		
China	-	-	-	01	18	34
Cyprus	-	-	-	-	-	01
India	10	05	-	02	01	
Iran	89	83	72	64	78	63
Iraq	02	01	02	01	03	02
Jordan	07	04	01	04	-	01
Kuwait	02	01	-	01	-	01
Kenya	-	-	-	-	-	01
Malaysia	01	01	04	01	09	03
UAE	-	02	03	02	-	-
Nigeria	13	21	13	10	09	-
Nepal	01	01	-	-	-	-
Oman	03	-	01	01	-	01
Palestine	02	04	01	02	-	-
Pakistan	81	61	70	56	93	65
Poland	01	-	-	-	-	-
Russia	01	-	-	-	-	-
Saudi Arabia	08	05	09	11	06	16
South Africa	-	-	02	03	02	01
South Korea	-	-	02	01	02	01
Sudan	-	-	-	01	-	-
Thiland	-	-	02	-	-	-
Taiwan	-	-	-	-	02	02
Turkey	03	02	24	34	74	37
UK	04	05	02	02	05	01
Sri Lanka	-	01	-	-	-	
UAE	-	-	-	02	01	01
Uganda	-	-	-	-	-	01
USA	02	01	-	-	-	-
Total	236	203	214	205	307	236

Practicing Editorial Triage, we communicate the decision to those authors within a week whose submissions cannot be accepted for further processing and the reasons are also communicated to them. The idea is that they can submit their manuscripts’ to some other journal without wasting any further time. We have also been doing publication audit for the last few years ^6^^-^^8^ to look at our strength and weaknesses which helps us to think and implement intervention strategies to overcome our shortcomings and plan for future. A careful look at the Year 2012 shows that the number of submissions continued to increase from 931 in the Year 2011 to 1023 in 2012. There has been significant increase in submissions from countries like China, Saudi Arabia and Turkey which also shows the pace of research in these countries. (Table-1) Number of submissions slightly decreased from Iran and Pakistan because of the strict peer review system that we practice. The number of manuscripts published during 2012 also decreased from 307 in the Year 2011 to 236 in the Year 2012, again because we were a bit stricter and only those manuscripts were preferred for publication which were of good quality and also had a chance of further citation. A total of 627 new submissions were not accepted for further processing (Table-II).Only selected Case Repots were accepted, 28 were rejected because of plagiarism while 16 were withdrawn by authors.(Table-III) As regards submissions from within the country, maximum number came from Karachi 78 followed by Lahore 23. (Table-IV) Since most of the medical institutions in Pakistan have now started publishing their own journals, hence we did not notice any increase in submissions. Largest category of published manuscripts were original articles 163 followed by case reports 48. (Table-V)

**Table-IV:** Manuscript received from Pakistan (2011-2012)

*City*	*2011*	*2012*
Abbottabad	2	-
Attock	-	1
Azad Kashmir	1	-
Bahawalpur	6	4
Bannu	-	1
Dera Ismail Khan	2	-
Faisalabad	4	3
Hyderabad	35	14
Islamabad/Rawalpindi	21	22
Jacobbabad	-	1
Karachi	78	78
Khairpur	1	-
Kohat	1	-
Lahore	23	23
Multan	10	3
Nawabshah	5	-
Peshawar	14	16
Quetta	1	2
Sialkot	1	-
Tando Alla Yar	-	1
Wah Cantt.	-	1
Total	205	170

**Table-V:** Category of Manuscripts Published during 2012

Editorial	4
Guest Editorial	1
Original Article	163
Case Reports	48
Brief Communications	11
Clinical Case Series	1
Correspondence	1
Review Article	4
Congress Proceedings	2
View Point	1
Total	236

 During the Year 2012 we also published a special issue for Isfahan University of Medical Sciences showcasing the research being done by their faculty members with a view to help Iranian research scientists to publish their work. The main objective was to promote regional cooperation through medical journalism besides promoting the art of scientific publishing in the region. ^9^

 Plans for the Year 2013 include increasing the frequency of publication of the journal from quarterly to Bimonthly, arranging for Digital Object Identified (DOI) number for all the manuscripts which are published in the journal, arrange generation of XML files of the manuscripts’ so that its full text availability on PubMed Central could be ensured. Earlier Pakistan Journal of Medical Sciences was approved by PubMed Central for inclusion but we could not arrange the XML files for which now arrangements are being made. Hopefully if all goes well, all the above objectives will be achieved during the current year and then efforts will be made to get it indexed in Medline as well.

 We practice Open Peer Review policy. During 2012 there were two instances when the manuscripts submitted by two PhD scholars were sent for review, the reviewers said that despite the fact that names of some medical heavy weights were included as authors, the studies suffered from many important flaws and asked for further guidance. They were assured to ignore the names of heavy weights and go ahead with the peer review and point out the deficiencies, give suggestions as to how the manuscripts can be improved further. This encouraged the Reviewers, who then did a commendable job; their comments were conveyed to the authors who revised their manuscripts which were eventually accepted for publication. The duties and responsibilities of Editors are just not to reject the papers but also help and guide the reviewers to perform their duties keeping up the professional ethics. Actually what happens is that most of these big names including institution heads, who act as Supervisors are too busy, they seldom have time to guide these researchers but at the same time are also keen to have their names included as authors. The Editors have to be careful of these “show-pieces” and follow publication ethics which of course can have its own repercussions. But even then the Editors are supposed to have no mercy for “chronic offenders” who indulge in scientific misconduct. At the same time there is no need to help the authors which are keen to see their publications in print the very next day.^10^ They often try to convince the editors that they already have many papers to their credit published in very high Impact Factor journals; hence they would try their best to pressurize the editors to accommodate them. They need to be informed that peer review takes some time and there is no short cut.^10^ At the same time authors digging up old study and republishing it with some new outcome “terrible research” and those following their own earlier experiments, plan to publish putting a new substance is “pointless research” all of which does not need to be published.

 We received Impact Factor a few years ago and at present it is 0.161 for 2012. IF is considered one of the important (but not the only one) measures to judge the quality and standard of a journal though at times it is too much over emphasized. It has its own limitations and drawbacks. Though we have had a successive increase in citations from Pakistan Journal of Medical Sciences over the year’s i.e. 198 in 2009, 275 in 2010 and 283 in 2011 but our Impact Factor decreased from 0.203 in 2009 to 0.161 in 2012 simply because we published more articles. This declining Impact Factor, one must admit, has been one of the factors in rejection of a large number of submissions because we do not want our Impact Factor to decrease further, though the number of submissions has also increased every year. Playing this Impact Facto game is a very tricky business but we do not wish to be harsh with the authors and would continue to follow the author friendly policy with the objective of promoting research culture in the country and the region. On the whole we feel satisfied at our performance in the preceding year and pledge to utilize all possible resources, opportunities to improve the quality of manuscripts’ accepted for publication thereby raising the standard of the Journal.
